# Electronic excitation induced amorphization in titanate pyrochlores: an *ab initio* molecular dynamics study

**DOI:** 10.1038/srep08265

**Published:** 2015-02-09

**Authors:** H. Y. Xiao, W. J. Weber, Y. Zhang, X. T. Zu, S. Li

**Affiliations:** 1 School of Physical Electronics, University of Electronic Science and Technology of China, Chengdu 610054, China; 2 Department of Materials Science & Engineering, University of Tennessee, Knoxville, TN 37996, USA; 3 Materials Science & Technology Division, Oak Ridge National Laboratory, Oak Ridge, TN 37831, USA; 4 Institute of Fundamental and Frontier Sciences, University of Electronic Science and Technology of China, Chengdu 610054, China; 5 School of Material Science and Engineering, University of New South Wales, Sydney, 2052, Australia

## Abstract

The response of titanate pyrochlores (A_2_Ti_2_O_7_, A = Y, Gd and Sm) to electronic excitation is investigated utilizing an *ab initio* molecular dynamics method. All the titanate pyrochlores are found to undergo a crystalline-to-amorphous structural transition under a low concentration of electronic excitations. The transition temperature at which structural amorphization starts to occur depends on the concentration of electronic excitations. During the structural transition, O_2_-like molecules are formed, and this anion disorder further drives cation disorder that leads to an amorphous state. This study provides new insights into the mechanisms of amorphization in titanate pyrochlores under laser, electron and ion irradiations.

The response of complex ceramics to extreme environments is of great interest for a broad range of applications. Ceramics for nuclear applications are exposed to high radiation environments that can lead to microstructure evolution, phase transitions, and degradation of physical and chemical properties. Ions, electrons and intense laser pulses can be used for precision micromachining, forming nanostructures, processing thin films, and inducing phase transitions. Pyrochlore-structured oxides, with the general formula A_2_B_2_O_7_ (A = Y or another rare earth element; B = Ti, Zr, Sn or Hf)^1^,^2^, represent a broad class of complex ceramics that exhibit an enormous range of physical, chemical and electric properties, including high ionic conductivity, superconductivity, luminescence, and ferromagnetism^3^. This leads to their significant potential use in a wide range of technical applications, such as hosts for oxidation catalysts, solid electrolytes in high temperature fuel cells, and ceramic thermal barrier coatings^4^,^5^,^6^. Modifying properties and creating new functionalities in pyrochlore thin films, single crystals and bulk materials often involves electron, ion or pulsed laser irradiation. Chemically durable pyrochlores are also candidate host matrices for the immobilization of plutonium and minor actinides that are generated through reprocessing of spent fuel from nuclear reactors^3^,^7^,^8^,^9^.

During the past decade, there have been many studies of ion-irradiation effects in pyrochlores^10^,^11^,^12^,^13^,^14^,^15^,^16^, and excimer laser irradiation has been employed to process pyrochlore thin films^17^, induce phase transitions^18^, and embed pyrochlore nanostructures^19^. Some pyrochlores undergo a structural phase transition from ordered pyrochlore to an amorphous structure due to self-radiation from actinide decay^20^ or external ion irradiation^21^. For example, amorphization was observed in the pyrochlore Gd_2_Ti_2_O_7_ due to self-radiation damage from α-decay of incorporated ^244^Cm at room temperature^20^,^22^. Lian et al. have investigated the irradiation response of A_2_Ti_2_O_7_ (A = Sm to Lu, and Y) pyrochlores by 1-MeV Kr ions at temperatures from 293 to 1073 K, and found that most of the titanate pyrochlores are readily amorphized^23^. Several possible mechanisms, such as direct-impact (in-cascade) amorphization within an individual collision cascade, the local accumulation of high defect concentrations due to the overlap of collision cascades, or a combination of these processes have been proposed to explain the origin of irradiation-induced amorphization^24^,^25^. These mechanisms are mainly based on atomic collision processes caused by energy transfer from energetic charged particles to atomic nuclei. Besides this effect, electronic excitation or ionization arising from the electronic energy loss of energetic ions may also weaken local atomic bonds and cause the collapse of the crystalline phase and structural amorphization at temperatures below the melting point^26^,^27^. In recent years, several swift heavy ion irradiation investigations have been carried out on pyrochlores to study the effects of intense electronic excitation^28^,^29^,^30^,^31^,^32^,^33^,^34^. The large local electronic excitation that occurs along an individual ion path leads to a thermal spike that creates an amorphous track in titanate pyrochlores^30^, and full amorphization results from the overlapping of these tracks^32^. Moll et al. compared the structural transitions in Gd_2_Ti_2_O_7_ single crystals irradiated with high- and low-energy heavy ions (870-MeV Xe and 4-MeV Au), and suggested that at high energy the structural amorphization is dominated by high electronic excitation, while at low energy the amorphization is driven by ballistic nuclear energy deposition from the ions^35^. More recently, Thomé et al. observed that ionization from 36-MeV W ions contributes to amorphization in Gd_2_Ti_2_O_7_^36^, although the mechanism for the ionization-induced amorphization was not identified.

Experimentally, electron beam irradiation using transmission electron microscopy is a direct technique to simulate the effects of electronic excitation and ionization from β-particles and γ-rays in reasonable laboratory time periods^37^,^38^. This technique has been employed to study the electronic effects on preamorphized SrTiO_3_ and Sr_2_Nd_8_(SiO_4_)_6_O_2_, and electron-beam-induced recrystallization was observed^39^,^40^,^41^, demonstrating that electronic excitation or ionization from electron irradiation can have substantial effects on the structure of materials. This raises the question of the role of electronic excitation at lower ion energies on the irradiation-induced crystalline-to-amorphous transition in pyrochlores, i.e., whether electronic excitation contributes to the amorphization or not. What will happen if only a small fraction of electrons in pyrochlores is excited? Theoretically, simulation of electronic excitation effects is a challenging task, since the dynamics of electron-hole recombination should be considered, and new computational techniques are needed to describe this effect^41^. In this study, an *ab initio* molecular dynamics (AIMD) method^42^,^43^ is employed to explore how low electronic excitation influences microstructural evolution in titanate pyrochlores. It is shown that at room temperature even ~2 % electronic excitation can induce structural amorphization, with the formation of O_2_-like molecules during the amorphization process. This mechanism is found to be different from that of intense electronic excitation induced by swift heavy ions irradiation, where local melting from a thermal spike leads to a quenched melt structure^30^. Our calculations suggest that electronic excitation may contribute to structural amorphization of pyrochlores under low or medium energy electron and ion irradiation. While intense electron and pulsed laser irradiation studies on Gd_2_Ti_2_O_7_ and other rare-earth titanate pyrochlores have not yet reported, the results of the present study provide some guidance on what might be expected.

## Computational Details

All the calculations were carried out within the density functional theory (DFT) framework using the projector augmented wave method, as implemented in the Vienna Ab Initio Simulation Package (VASP)^44^. Projector augmented-wave pseudopotentials^45^ were used to describe the interaction between ions and electrons, and the exchange-correlation effects were treated using the generalized gradient approximation (GGA) in the Perdew-Wang parameterization. The valence electronic configurations are 6*s*^2^5*p*^6^5*d*^1^ for Gd, 4*s*^2^4*p*^6^5*s*^1^4*d*^2^ for Y, 5*s*^2^6*s*^2^5*p*^6^5*d*^1^ for Sm, 4*s*^1^3*d*^3^ for Ti and 2*s*^2^2*p*^4^ for O. Computations were based on a supercell consisting of 88 atoms, with a 1 × 1 × 1 Monkhorst-Pack^46^ k-point sampling in the Brillouin zone and a cutoff energy of 400 eV for the basis set. The calculated lattice parameters are a_0_ = 10.11 Å, *x*_O48f_ = 0.3301 for Y_2_Ti_2_O_7_, a_0_ = 10.22 Å, *x*_O48f_ = 0.3292 for Gd_2_Ti_2_O_7_, and a_0_ = 10.31 Å, *x*_O48f_ = 0.3269 for Sm_2_Ti_2_O_7_, which agree well with the experimental results and other calculations^5^,^23^. The crystal was first equilibrated for 2 ps at 100 K. To study the response of titanate pyrochlores to electronic excitation, we simplified the simulation by removing several electrons from high-lying valence band states and a jellium background was used to compensate for the loss of charge due to electron removal. Selection of the electrons that should be removed was weighted according to the orbital energies. This method has been suggested and validated by Li et al. in studies of the role of electronic excitation in the amorphization of Ge-Sb-Te alloys^47^. The excitation concentration, which is defined as the ratio of the number of excited valence electrons to the number of total electrons, varies from 0 to 2.3 %. Since the supercell contains 1824 electrons for Gd_2_Ti_2_O_7_, 1424 electrons for Y_2_Ti_2_O_7_ and 1792 electrons for Sm_2_Ti_2_O_7_, the maximum excitation concentration of 2.3 % correspond to 42, 33 and 41 excited electrons, respectively. Under laser beam irradiation, the laser fluence at 400 nm for 1 % excitation in Gd_2_Ti_2_O_7_ is estimated to be about 9.5 × 10^2^ − 5.4 × 10^3^ mJ/cm^2^ (see supplementary material). *Ab initio* MD calculations were then performed to completely relax the whole system until it reached an equilibrium state at temperatures of interest. Subsequently, the removed electrons were placed back to mimic the recombination of electrons and holes. The AIMD simulation was conducted with a time step of 3 fs and a isothermal-isochoric ensemble, in which the Nosé-Hoover thermostat was employed to control the temperature. The simulation times are 12 ps and 3 ps for electronic excitation and electron-hole recombination, respectively.

## Results and Discussion

**Figure 1:**
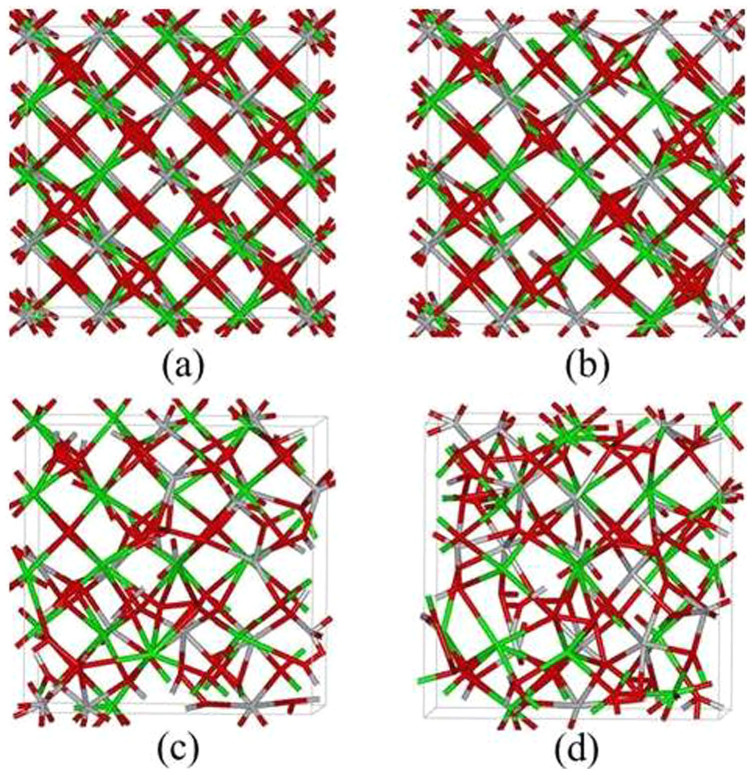
Geometrical configuration of Gd_2_Ti_2_O_7_ with excitation concentration of (a) 0.0 %; (b) 0.99 %; (c) 1.64 % and (d) 2.30 % at 300 K. The green, grey and red sticks represent Gd, Ti and O, respectively.

**Figure 2:**
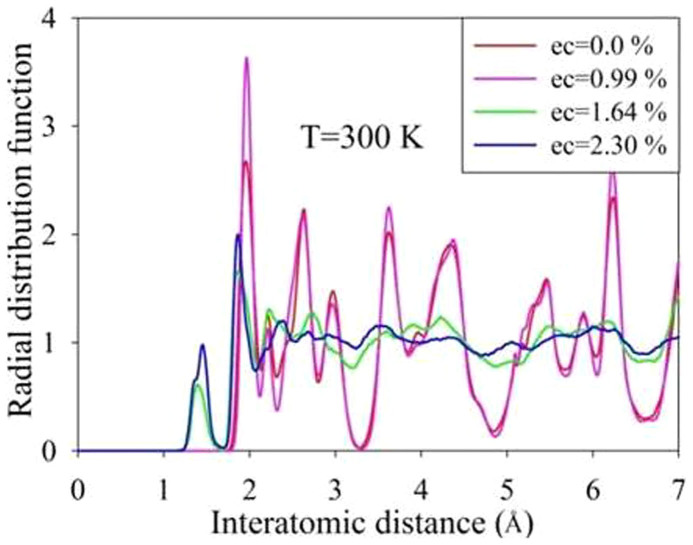
Radial distribution functions as a function of excitation concentration for Gd_2_Ti_2_O_7_ at 300 K.

**Figure 3:**
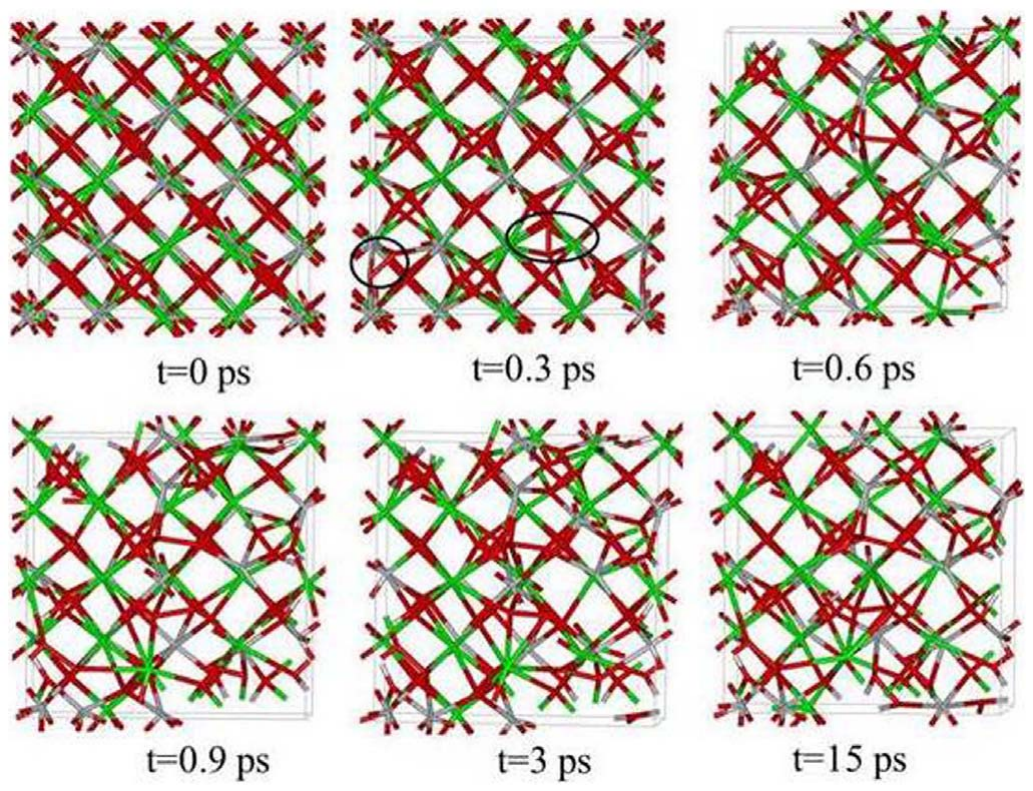
Structural evolution of Gd_2_Ti_2_O_7_ with 1.64 % excitation at 300 K. O_2_-like molecules are marked by the black circles.

**Figure 4:**
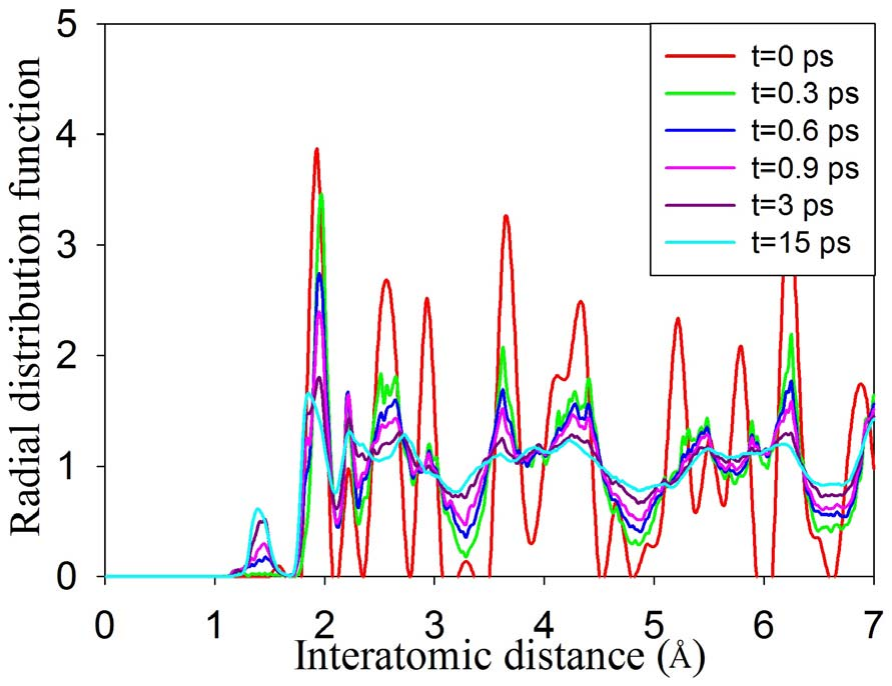
Radial distribution functions as a function of time for Gd_2_Ti_2_O_7_ at 300 K.

AIMD simulations were first carried out on Gd_2_Ti_2_O_7_ at 300 K with the concentration of excited electrons being 0.0 %, 0.99 %, 1.64 % and 2.30 %. The results in Fig. 1 show the final geometrical configurations of Gd_2_Ti_2_O_7_ for different excitation concentrations. The pyrochlore structure remains ordered when no electrons are excited, and the ordered structure changes very slightly for 0.99 % excitation. However, significant structural changes occur for 1.64 % and 2.30 % excitation. Atomic coordination analysis shows that the Gd and Ti atoms are eight- and six-coordinated with oxygen atoms in the ideal pyrochlore structure; whereas for 1.64 % excitation, the average coordination numbers are lowered to 4.6 and 4.5 for Gd and Ti, respectively. The radial distribution function (RDF), shown in Fig. 2, is a measure of the structural order as a function of excitation concentration for Gd_2_Ti_2_O_7_ at 300 K. A material is amorphous when it retains short-range order but has lost long-range order. The results in Fig. 2 clearly show that the structures are ordered at short-range distances and disordered at long-range distances for 1.64 % and 2.30 % excitation, suggesting that 1.64 % and 2.30 % electronic excitation can induce a crystalline-to-amorphous transition in Gd_2_Ti_2_O_7_ at 300 K. Snapshots of structural evolution with 1.64 % excitation are illustrated in Fig. 3 and the corresponding RDFs are presented in Fig. 4. It is shown that structural amorphization starts at t = 0.3 ps, and the structure is completely amorphized at t = 3 ps.

**Figure 5:**
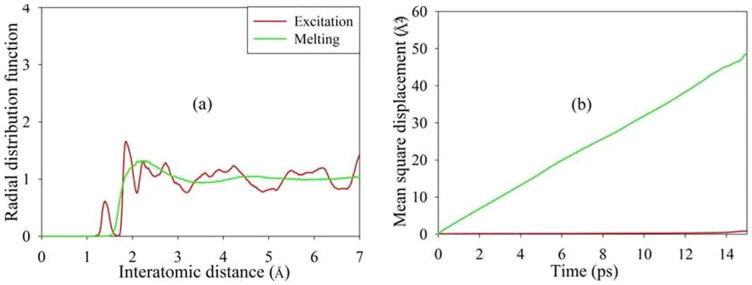
Comparison of (a) radial distribution functions and (b) mean square displacement between excited and melted Gd_2_Ti_2_O_7_.

In order to explore if the structural amorphization is a solid-liquid transition, the melting point of Gd_2_Ti_2_O_7_ was calculated by annealing a high-temperature melt state at lower temperatures until the free energy starts to decrease and is accompanied by recrystallization^47^. The melting point was determined to be ~1875 K, which is ~100 K lower than the experimental value of ~1973 K^48^. AIMD simulations without electronic excitation were then performed at 2000 K. Comparison of the RDF and mean square displacement (MSD) between the excited and melted Gd_2_Ti_2_O_7_ are shown in Figs. 5(a) and (b). It is noted that excitation-induced amorphization exhibits features significantly different from those of melt state. In the latter case, the structure is liquid and the MSD is ~50 times larger. These results indicate that electronic excitation-induced amorphization is a solid-solid transition rather than a solid-liquid transition.

**Figure 6:**
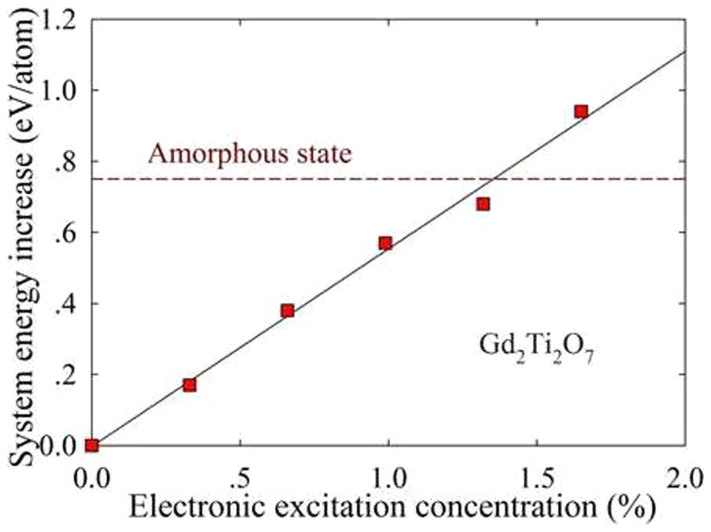
System energy increase in Gd_2_Ti_2_O_7_ as a function of electronic excitation concentration.

Figure 6 shows that the system energy increases with increasing excitation concentration and becomes more and more close to that of the liquid amorphous state. Above an excitation concentration of 1.36 %, the system energy for Gd_2_Ti_2_O_7_ is even larger than that for the melt state. These results indicate that, at higher excitation concentration, the energy barrier for structural amorphization decreases and the crystalline-to-amorphous transition is much easier to occur.

Irradiation damage effects of titanate pyrochlores have been investigated experimentally over a broad range of ion irradiation energy. Under 1-MeV Kr ion irradiation, where the energy loss to ionization is much larger than the damage energy going into atomic displacements, it was observed that all the titanate pyrochlores are easily amorphized under irradiation^23^. Swift heavy ion irradiation, with 1.43-GeV Xe ions by Lang et al.^29^,^30^, with 100-MeV ions by Patel et al.^28^, with 780-MeV Kr ions, 870-MeV Xe ions, 940-MeV Pb ions^31^ and 119-MeV U ions by Sattonnay et al.^32^, have been carried out on the Gd_2_(Zr_x_Ti_1−x_)_2_O_7_ system, as well as a series of zirconate pyrochlores. These studies show that structural modification induced by high electronic excitation is chemical composition dependent. At intermediate ion energies, Thomé et al. observed amorphization in Gd_2_Ti_2_O_7_ due to ionization from 36-MeV W ions^36^.

**Figure 7:**
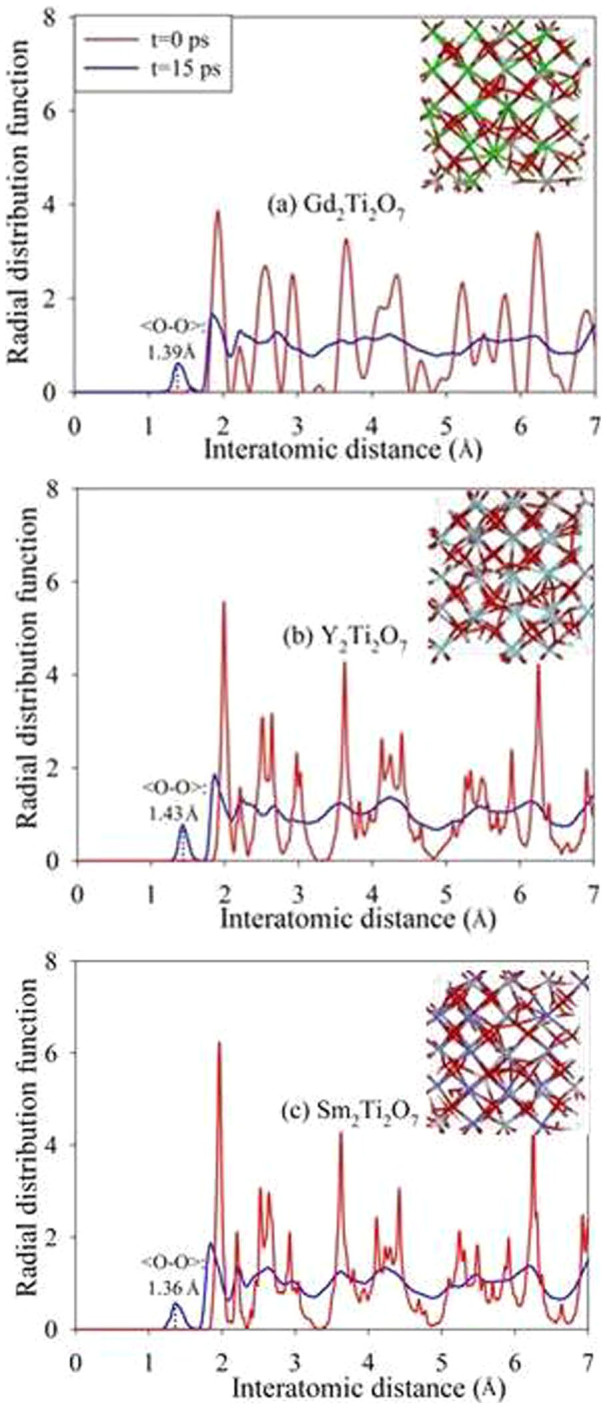
Comparison of radial distribution functions for titanate pyrochlores.

To investigate if low electronic excitation induced amorphization is a universal phenomenon in other titanate pyrochlores, AIMD simulations are also carried out on Y_2_Ti_2_O_7_ and Sm_2_Ti_2_O_7_, in which 2.11 % and 1.67 % electrons are excited at 300 K, respectively. The radial distribution functions of Y_2_Ti_2_O_7_ and Sm_2_Ti_2_O_7_ are compared with those of Gd_2_Ti_2_O_7_ with 1.64 % excitation at 300 K in Fig. 7. Similar to Gd_2_Ti_2_O_7_, both Y_2_Ti_2_O_7_ and Sm_2_Ti_2_O_7_ undergo a crystalline-to-amorphous structural transition, as indicated by the presence of short-range order and loss of long-range order in the RDFs. As the level of electronic concentration is relatively low and all the structural transition occurs at 300 K, these calculations suggest that titanate pyrochlores should be readily amorphized under local ionization rates that produce these levels electronic excitation. Ion irradiation of titanate pyrochlores (A_2_Ti_2_O_7_, A = Sm to Lu, and Y)^23^ with 1-MeV Kr ions, which have a high electronic to nuclear stopping ratio, readily leads to amorphization, consistent with our calculations.

**Figure 8:**
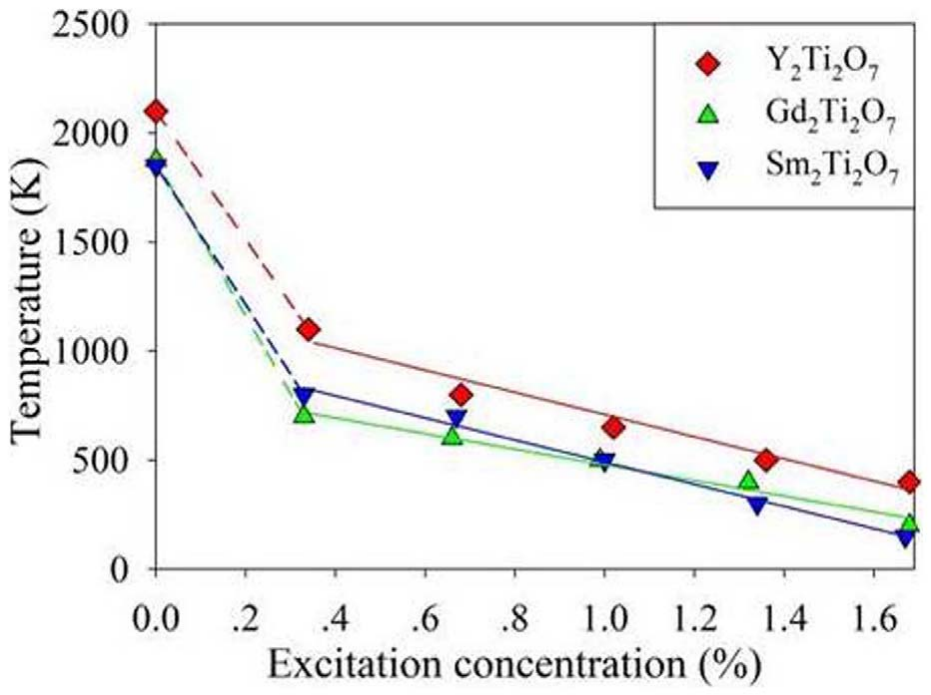
Transition temperatures of titanate pyrochlores as a function of excitation concentration. The calculated and fitted results are represented by symbols and solid lines, respectively.

**Figure 9:**
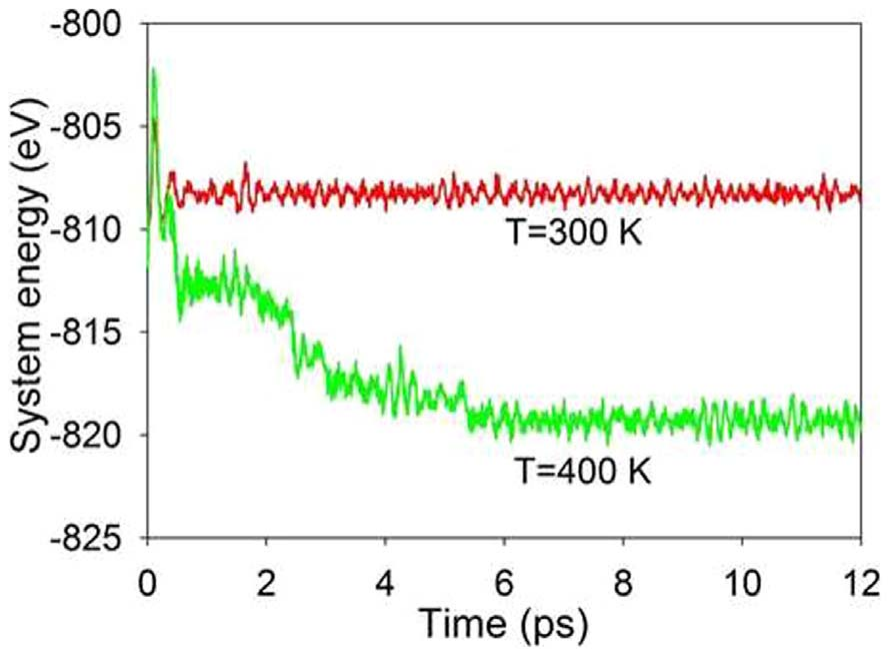
Variation of system energy for Gd_2_Ti_2_O_7_ (with ~1.32 % excitation) with time evolution at 300 and 400 K.

**Figure 10:**
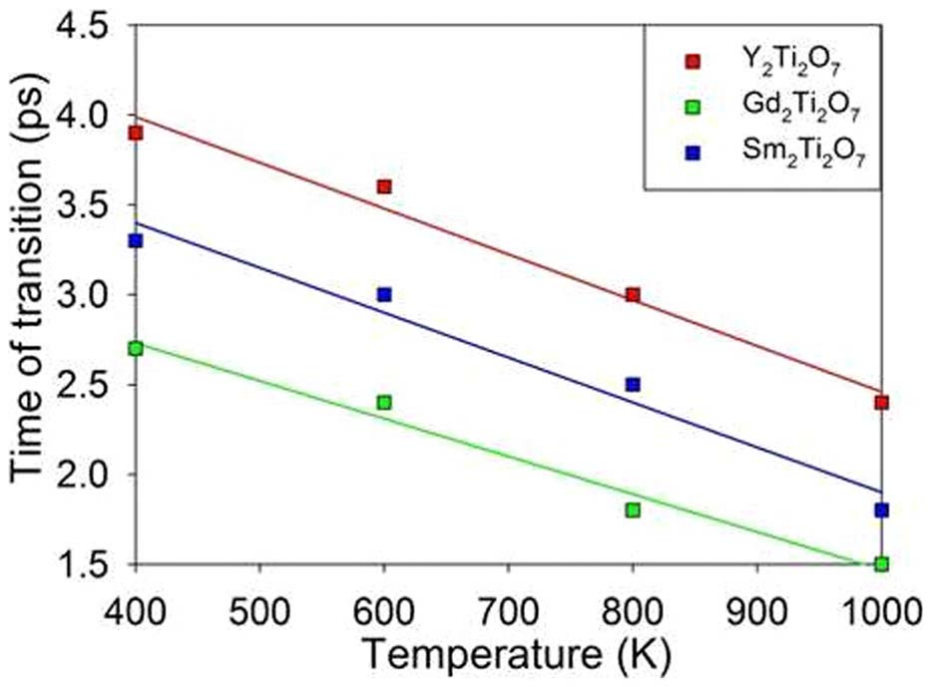
Time of transition for titanate pyrochlores as a function of temperature. The calculated and fitted results are represented by symbols and solid lines, respectively.

Lian et al. have reported that the irradiation response of titanate pyrochlores to ion irradiation depends on the size and electronic configuration of A-site cations, and Gd_2_Ti_2_O_7_ is the most easily amorphized^23^. To explore the difference in radiation resistance between the three titanate pyrochlores investigated here, we have explored how the excitation concentration of each compound influences its transition temperature. For each electronic concentration, the simulations were first performed at 100 K. If no structural transition occurs, the simulation was restarted at higher temperature with a temperature increment of 100 K. The temperatures at which structural amorphization starts to occur are then determined as the transition temperature. The considered excitation concentration varies from 0.33 % to 1.68 %. The change in transition temperature with excitation concentration for each compound is shown in Fig. 8. The transition temperatures are found to depend on the concentration of excited electrons for all compounds, and the temperature decreases with increasing excitation concentration. We also find that the transition temperature for Y_2_Ti_2_O_7_ is higher than that of Gd_2_Ti_2_O_7_ and Sm_2_Ti_2_O_7_; therefore, within the considered range of excitation concentration, Y_2_Ti_2_O_7_ is relatively more radiation resistant. On the other hand, the transition temperature for Gd_2_Ti_2_O_7_ is comparable to that for Sm_2_Ti_2_O_7_, indicating a similar radiation resistance to electronic excitation. This is consistent with ion irradiation experiments, where Y_2_Ti_2_O_7_ is more resistant to amorphization than Gd_2_Ti_2_O_7_ and Sm_2_Ti_2_O_7_ under 1-MeV Kr ions irradiation^23^. In our simulation only electronic excitation is considered, whereas in ion irradiation experiments both atomic collision and electronic excitation may contribute to the structural amorphization. When the electronic concentration is lower than 0.33 %, the dependence of transition temperature on excitation concentration is not clear. The calculated melting temperatures of 1875 K for Gd_2_Ti_2_O_7_, 2100 K for Y_2_Ti_2_O_7_ and 1850 K for Sm_2_Ti_2_O_7_ are also presented in Fig. 8. Our calculations indicate that increasing electronic excitation can effectively reduce the melting temperature. It should be pointed out that, at each electronic concentration, the electronic excitation is simulated for 12 ps, which is adequate to ensure the system reaches an equilibrium state. Figure 9 shows variation of the system energy for Gd_2_Ti_2_O_7_ (with ~1.32 % excitation) with time at transition temperature of 400 K. The changes in system energy at 300 K, where crystalline-to-amorphous transition does not occur, are also presented. It is obvious that the system reaches equilibrium states within 12 ps at both temperatures. The time of transition for titanate pyrochlores (with ~1.64 % excitation) as a function of temperature is presented in Fig. 10. It is noted that in all cases the time of transition decreases with increasing temperature. At 400 K the crystalline-to-amorphous transition time is predicted to be only 3.9, 3.3 and 2.7 ps for Y_2_Ti_2_O_7_, Gd_2_Ti_2_O_7_ and Sm_2_Ti_2_O_7_, respectively, indicating that the transition is very fast. Unfortunately, no experimental data are available in the literature to validate such processes.

**Figure 11:**
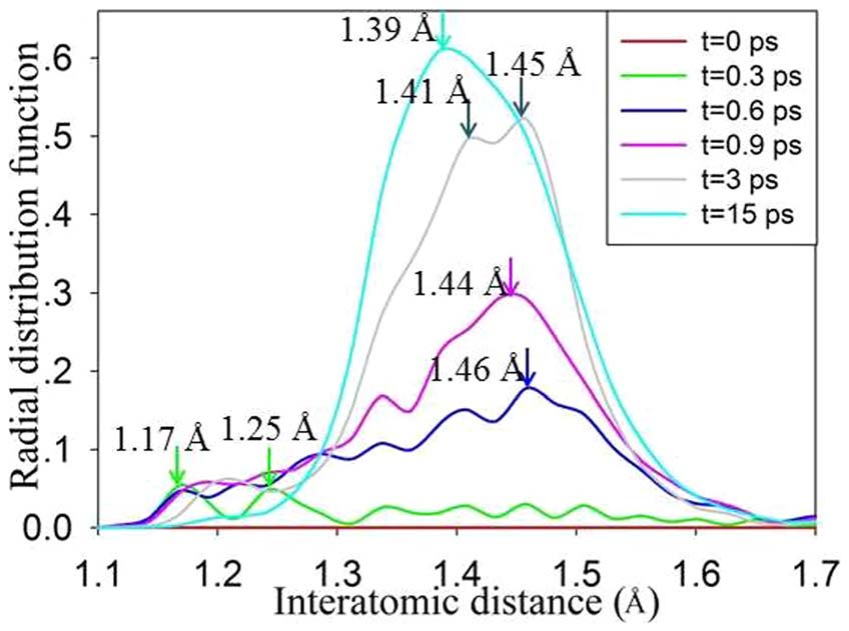
Radial distribution function for <O-O> bond in Gd_2_Ti_2_O_7_ (with 1.64 % excitation at 300 K).

Comparing the RDF for crystalline and amorphous states, as shown in Fig. 7, we find the most striking feature for the amorphous state is that a new <O-O> peak forms upon structural amorphization. Fig. 11 illustrates the radial distribution function for <O-O> bond in Gd_2_Ti_2_O_7_ (with 1.64 % excitation at 300 K). It is shown that with time evolution more and more <O-O> chemical bonds are formed and the bonding distance of 1.17 ~ 1.46 Å is close to that of an oxygen molecule, i.e., 1.24 Å, suggesting that O_2_-like molecules are formed during the amorphization process. Similar phenomena have been observed for Y_2_Ti_2_O_7_ and Sm_2_Ti_2_O_7_, for which the average <O-O> bonding distances at 15 ps are 1.43 and 1.36 Å, respectively. In Fig. 3, we note that, at t = 0.3 ps, the <O-O> bonds appear, which are marked by the circle, while other chemical bonds, including <Gd-O>, <Ti-O> and <Gd-Ti> bonds, remain nearly unchanged. With further time evolution, the disordering of other chemical bonds occurs, and complete amorphization takes place at t = 3 ps. These results indicate that anion disorder and cation disorder do not occur concurrently under these conditions.

**Figure 12:**
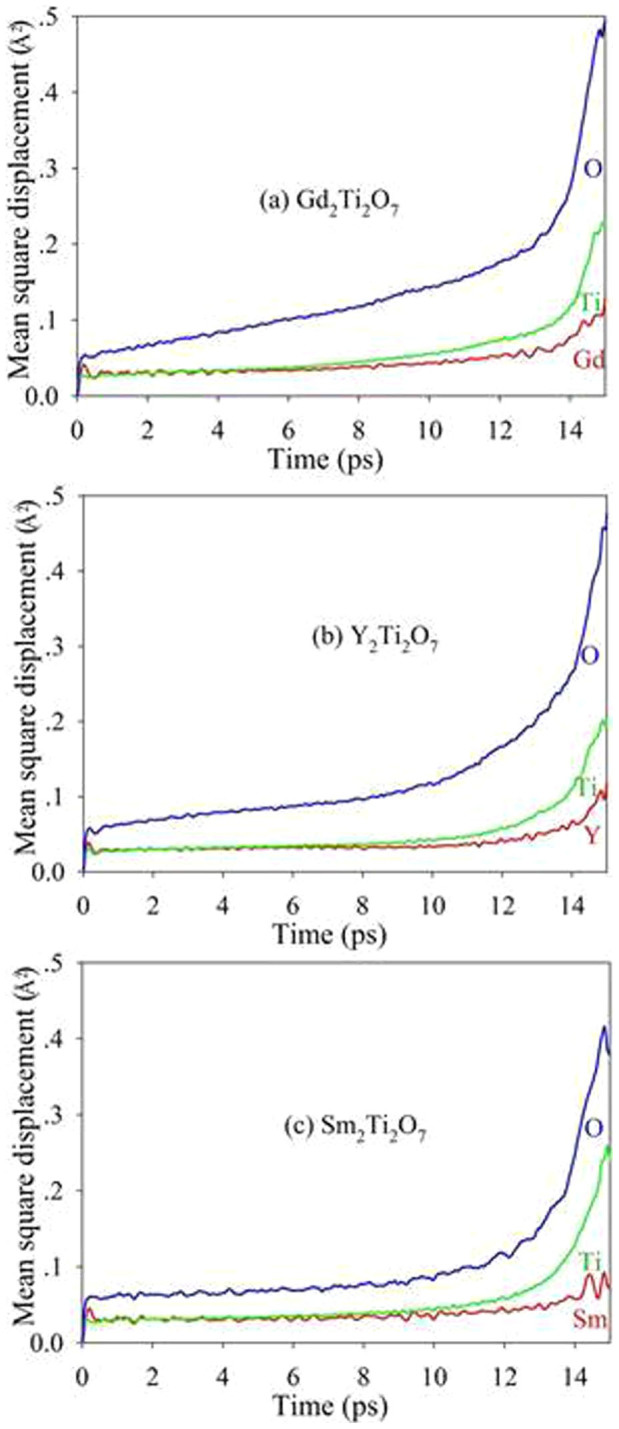
Mean square displacements of titanate pyrochlores.

**Figure 13:**
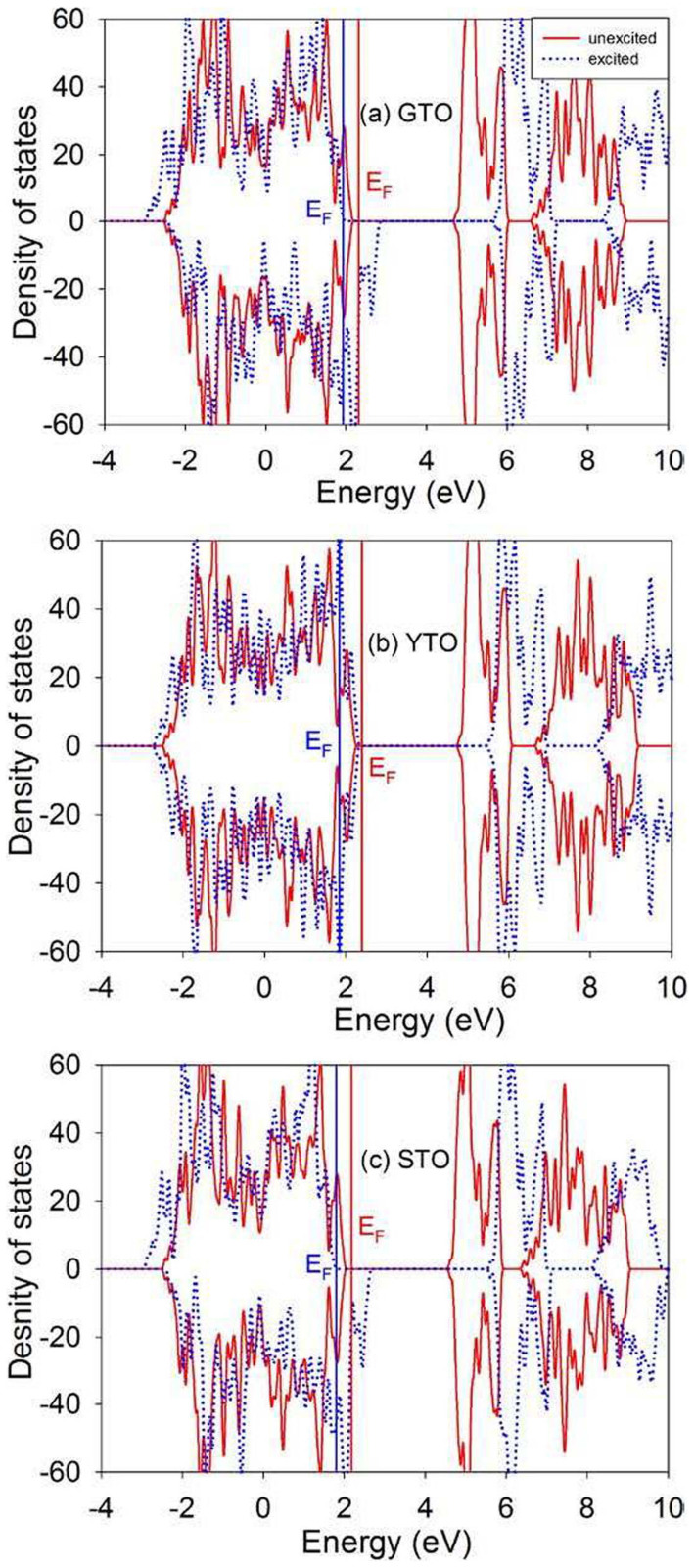
Total density of states distribution for Gd_2_Ti_2_O_7_ (GTO), Y_2_Ti_2_O_7_ (YTO) and Sm_2_Ti_2_O_7_ (STO) without and with electronic excitation (~1.64 %, no structural relaxation).

**Figure 14:**
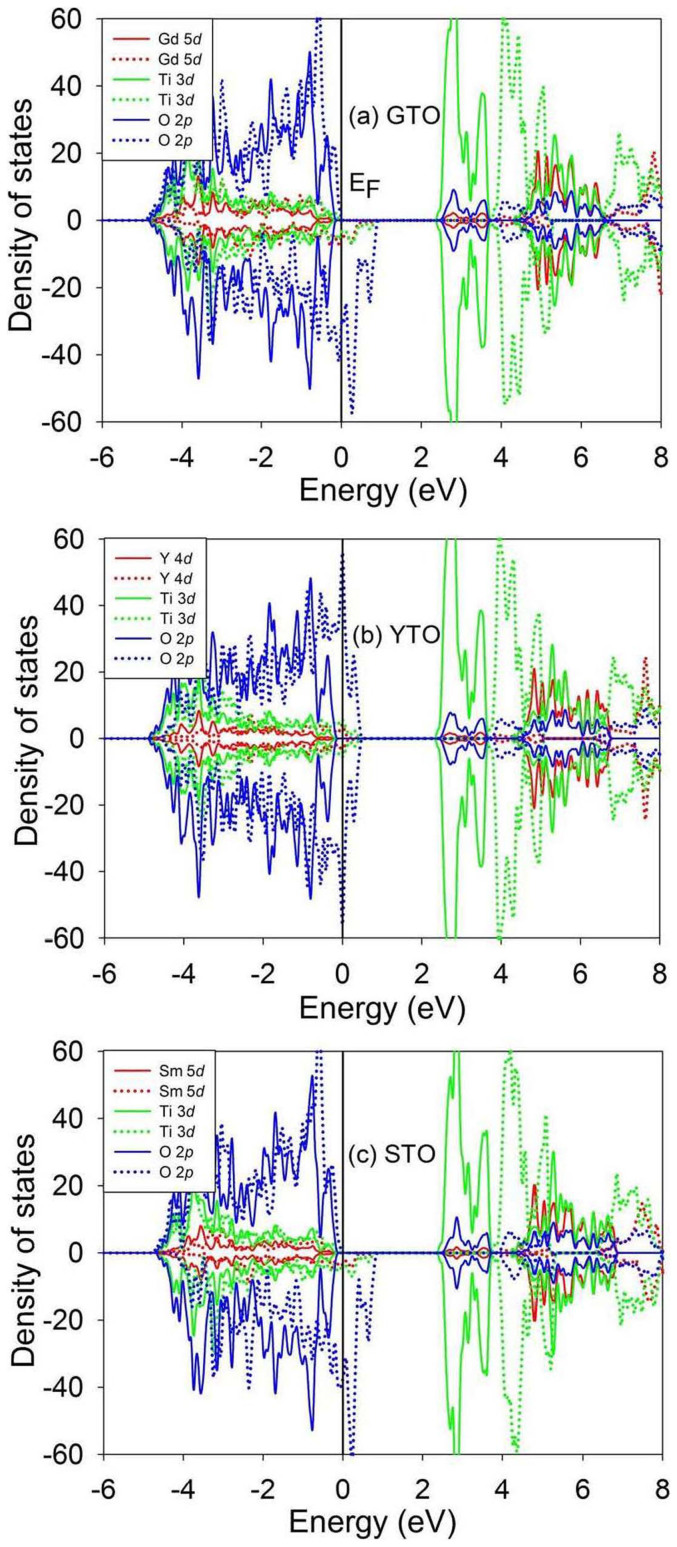
Projected density of states distribution for Gd_2_Ti_2_O_7_ (GTO), Y_2_Ti_2_O_7_ (YTO) and Sm_2_Ti_2_O_7_ (STO) without and with electronic excitation (~1.64 %, no structural relaxation).

**Figure 15:**
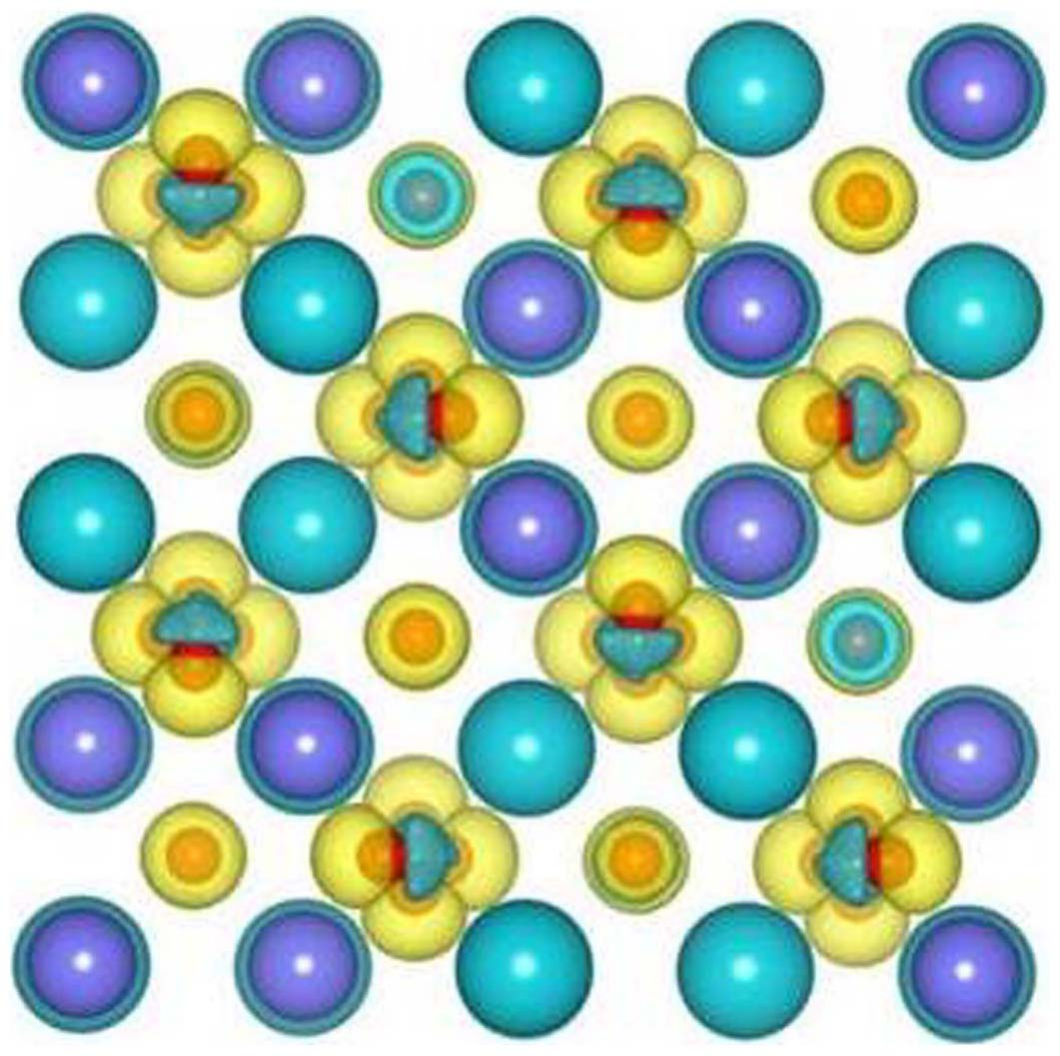
Spin density distribution for Gd_2_Ti_2_O_7_ with ~1.64 % excitation (without relaxation).

The MSDs for A, Ti and O in A_2_Ti_2_O_7_ (A = Gd, Y and Sm) are presented in Fig. 12. It is shown that, in all the compounds, the mean square displacements for oxygen are considerably larger than those for other chemical elements, i.e., the displacement of oxygens contributes mostly to the amorphization of pyrochlores. The total density of states for titanates without and with electronic excitation (~1.64 %, no structural relaxation) are shown in Fig. 13. It is noted that electronic excitation results in shifts of the Fermi energy to lower energy levels, exhibiting p-type doping character. Moreover, electrons are redistributed around the Fermi level. The results in Fig. 14 illustrate the projected density of states for titanates without and with electronic excitation (~1.64 %, no structural relaxation). We find that the valence band maxima are mainly contributed by O 2*p* orbitals hybridized with A 4*d* or 5*d* states and Ti 3*d* states, and a large number of O 2*p* electrons occupy the Fermi level. It is indicated that the valence electrons located at O 2*p* orbitals are more readily to be excited when the transferred energy is large enough. Figure 15 shows the spin density distribution for excited Gd_2_Ti_2_O_7_ without structural relaxation. It is found that the charges, which are indicated by the yellow sphere, are mainly located on oxygen atoms. These results suggest that upon electronic excitation most of O 2*p* electrons are excited, leading to charge redistribution and changes in interatomic potentials, which causes anion disorder and the formation of O_2_-like molecules. Subsequently, cation disorder is induced, and both anion and cation disorders drive the structure towards the amorphous state. Experimentally, Lian et al. also suggested that, under ion irradiation, anion disorder precedes cation disorder in Gd_2_Ti_2_O_7_, Er_2_Ti_2_O_7_ and Lu_2_Ti_2_O_7_^49^.

The amorphization mechanism revealed in this study is different from that for the intense local electronic excitation caused by swift heavy ions irradiation^32^. Irradiations with high energy heavy ions, such as 1.43-GeV Xe ions and 119-MeV U ions, have been carried out on Gd_2_Ti_2_O_7_ by Lang et al.^29^,^30^, Patel et al.^28^, and Sattonnay et al.^31^,^32^ In these studies, the electronic stopping power is orders of magnitude higher than the nuclear stopping power, and the highly excited electrons create a local thermal spike due to electron-phonon coupling that causes local thermal melting^50^. The quenching of this thermal melt leads to the creation of cylindrical damaged regions, i.e., ion tracks. The formation and the overlapping of individual ion tracks^31^ eventually results in the crystalline-to-amorphous transition in pyrochlores^32^. In our simulation, only a small concentration of electrons is excited, and there is a lack of the kinetic energy given to electrons as in the case of swift heavy ions. The results of the current study more closely simulate the irradiation with low-energy electrons, light ions, or pulsed lasers; however, it may not be easy to achieve the level of electron excitation needed to induce amorphization, without intense beams that can cause local heating. While lower levels of electronic excitation may not directly lead to amorphization at relatively low temperature, they may contribute to disordering and amorphization during atomic collision processes by destabilizing the structure, for example through broken bonds and increased energy states. The results of this study should encourage carefully designed experimental investigations to validate the predictions.

## Conclusions

In summary, an *ab initio* MD method has been employed to study the structural stability of Y_2_Ti_2_O_7_, Gd_2_Ti_2_O_7_ and Sm_2_Ti_2_O_7_ under electronic excitation. It is shown that a crystalline-to-amorphous structural transition can be induced in each of the titanate pyrochlores by electronic excitation. This electronic excitation-induced amorphization is a solid-solid transition rather than a solid-liquid transition. The temperature at which structural amorphization starts to occur has been determined for each compound with excitation concentration varying from 0.3 % to 2.11 %. For all compounds, this transition temperature depends on the concentration of excited electrons and decreases with increasing excitation concentration. The transition temperature for Y_2_Ti_2_O_7_ is higher than that of Gd_2_Ti_2_O_7_ and Sm_2_Ti_2_O_7_, and the transition temperatures for Gd_2_Ti_2_O_7_ and Sm_2_Ti_2_O_7_ are comparable to each other. When electronic excitation occurs, the excitation of O 2*p* electrons dominates, which causes anion disorder and the formation of O_2_-like molecules. Cation disorder is further induced by anion disorder, and both drive the structure towards the amorphous state. The mechanism for amorphization induced by electronic excitation in this study is different from that caused by the intense local electronic excitation from swift heavy ions, which occurs by thermal melting. This study indicates the importance of electronic excitation in phase transition processes in pyrochlores under electron, ion and pulsed laser irradiations, and it provides insights on the use of intense ionizing irradiation for micromachining, surface modifications, and thin film processing.

## Supplementary Material

Supplementary Information Electronic excitation induced amorphization in titanate pyrochlores: an ab initio molecular dynamics study
