# Supplementary material for: Electronic excitation induced amorphization in titanate pyrochlores: an ab initio molecular dynamics study
Source: Sci Rep. 2015 Feb 9;5:8265. doi: 10.1038/srep08265 (PMC5389128; doi:10.1038/srep08265)
Supplement: Supplementary Information — Electronic excitation induced amorphization in titanate pyrochlores: an ab initio molecular dynamics study [file srep08265-s1.pdf]

Supplementary information

Electronic excitation induced amorphization in titanate pyrochlores: an *ab initio* molecular dynamics study

H.Y. Xiao,[a,*] W.J. Weber,[b,c] Y. Zhang,[c,b] X.T. Zu[a,d], S. Li[e]

[a]*School of Physical Electronics, University of Electronic Science and Technology of China, Chengdu 610054, China*

[b]*Department of Materials Science & Engineering, University of Tennessee, Knoxville, TN 37996, USA*

[c]*Materials Science & Technology Division, Oak Ridge National Laboratory, Oak Ridge, TN 37831, USA*

[d]*Institute of Fundamental and Frontier Sciences, University of Electronic Science and Technology of China, Chengdu 610054, China*

[e]*School of Material Science and Engineering, University of New South Wales, Sydney, 2052, Australia*

[*]Corresponding author. Tel.: +86 28 83202130.

E-mail address: hyxiao@uestc.edu.cn (H.Y. Xiao).

Under laser beam irradiation, the laser fluence at 400 nm for 1 % excitation in $Gd_2Ti_2O_7$ is about $9.5\times10^2 - 5.4\times10^3$ mJ/cm^2 .The intensity of the e-h pairs that are generated can be estimated by $N_{e-h} = \frac{(1-R)\times\alpha_{eff}\times F}{\hbar\omega_0}$ [S1], where F and ω_0 are

the laser fluence and frequency, R and α_{eff} are the reflectivity and effective absorption coefficient for the sample. Using $R(400\text{ nm}) = 10\% - 20\%$, $\alpha_{eff}(400\text{ nm}) = 2\times10^3 - 1\times10^4\text{ cm}^{-1}$, $\hbar\omega_0 = 3.1\text{ eV}$ [S2-S5], $N_{e-h} = 1\% \times N_{total} = 1\% \times 1.708\times10^{24}\text{ cm}^{-3}$, we thus obtain $F = 9.5\times10^2 - 5.4\times10^3\text{ mJ/cm}^2$.

[S1] Sokolowski-Tinten, K., Bialkowski, J.,Von der Linde, D. *Phys. Rev. B* **51**, 14 186 (1995).

[S2] Weller, M.T.,Hughes, R.W., Rooke, J., Knee, C.S., Reading, J. *Dalton Trans*. **19**, 3032 (2004).

[S3] Malkin, B.Z., Zakirov, A.R., Popova, M.N., Klimin, S.A., Chukalina, E.P., Antic-Fidancev, E., Goldner, Ph., Aschehoug, P., DhalenneMalkin, G. *Phys. Rev. B* **70**, 075112 (2004).

[S4] Raj, A.K.V., Rao, P.P., Sreena, T.S., Sameera, S., James, V., Renju, U.A. *Phys. Chem. Chem. Phys*., DOI: 10.1039/C4CP03311F, 2014

[S5] Pavlov, R.S., Marza, V.B., Carda, J.B. *J. Mater. Chem.*, **12**, 2825 (2012).
